# National science foundation grant awardees’ perspectives on Article X and sexual harassment in science

**DOI:** 10.1371/journal.pone.0300762

**Published:** 2024-04-30

**Authors:** Chithra R. Perumalswami, Amanda K. Greene, Kent A. Griffith, Reshma Jagsi

**Affiliations:** 1 Center for Bioethics and Social Sciences in Medicine, University of Michigan Medical School, Ann Arbor, MI, United States of America; 2 Winship Cancer Institute, Emory University, Atlanta, GA, United States of America; The Hong Kong Polytechnic University, HONG KONG

## Abstract

Federal scientific agencies seek to make an impact on the continued prevalence of sexual harassment in the scientific academic community beyond institutional self-regulation. The National Science Foundation’s Article X, released in 2018, is one of the most significant and ambitious federal policy initiatives to address sexual harassment. The present article presents the results of the first study to examine scientists’ knowledge and attitudes about this important recent policy. We found, although overall knowledge about Article X was fairly low, the majority of participants responded positively to it. Crucially, impressions of the policy varied based on past experience and demographic factors. Individuals who had experienced harassment in the past year were less likely to believe the policy would help reduce sexual harassment in the sciences compared to those who had not experienced harassment (OR = 0.47, 95% CI:0.23–0.97, p = .034) and had greater odds of endorsing that the policy failed to go far enough (OR = 2.77, 95% CI:1.15–6.66, p = .023). Associations between demographic factors and views of the policy were less pronounced, but it is notable that, compared to their White counterparts, Black participants were more likely to believe the policy went too far (OR = 5.87, 95% CI:1.04–33.17, p = .045). Additionally, concerns were raised about the institutional enforcement of these policies and the existence of sufficient protections for survivors. Our work has implications for NSF’s continued evaluation of the efficacy of this program as well as for other federal agencies implementing or considering similar policies.

## Introduction

### Sexual harassment in academia

A major cultural reckoning with the troubling prevalence of sexual harassment in the workplace across multiple sectors developed in the past decade. The explosion of the #MeToo movement in 2017 and the associated outpouring of sexual harassment stories on social media catalyzed both a heightened awareness of the enduring extent of this problem and targeted actions to address it [1]. Academia was not exempt from the #MeToo movement; social media and traditional news media both received many stories from university students and faculty that painted a bleak picture of the ivory tower [2–6]. This deluge of personal narratives was consistent with earlier reviews of workplace sexual harassment that had identified academia as second only to the military in terms of the severity of the issue [7].

Research confirms that sexual harassment remains pervasive in the scientific academic community [8–10]. Women are more likely than men to be the targets of harassment, and men are more likely than women to be the perpetrators [11–13]. Intersectionality also influences the risk and burden of sexual harassment in academic settings [14]. Notably, individuals with minoritized identities appear uniquely and disproportionately impacted [15]. For example, Black female faculty may experience sexual harassment differently than their white female counterparts and also find it compounded by concurrent racial harassment [11, 16].

### Federal response and responsibility

While many policies have been instated at the individual institution level to address sexual harassment in science, federal scientific agencies are well-situated to make an impact beyond institutional self-regulation. The National Academies of Science, Engineering, and Medicine (NASEM) published a landmark report on sexual harassment in June 2018, declaring it a national priority and issuing a call to action [17]. The report details how Title IX of the Education Amendments Act of 1972 and Title VII of the Civil Rights Act of 1964 led to unintended consequences of increased symbolic compliance at the institutional level but failure to adequately protect targets at the individual level in terms of preventing sexual harassment and mitigating its consequences after it occurs [17, 18]. The NASEM report publication and reception catalyzed a policy stream in which federal scientific agencies have proposed policy changes with regards to the impact of sexual harassment on science [19, 20]. Given that many institutions of higher education where gender harassment, unwanted sexual attention, and coercion transpire receive billions of dollars in funding from federal scientific agencies, this funding has been identified as a key lever of change to reduce the culture of sexual harassment in science [21–23].

One of the most significant and ambitious initiatives in this area has been the National Science Foundation’s (NSF) Article X, published in the Federal Register on October 21, 2018 [24]. Article X seeks to leverage NSF’s influence (and control of a substantial $8 billion in grant funding in 2019) to contribute to efforts to address the problem of persistent and pervasive sexual harassment in the sciences. It provides clear potential consequences for NSF grant awardees who have been found responsible for harassment, including substitution or removal of the principal investigator (PI) or co- principal investigator (Co-PI) from the award, reduction in the award funding amount, suspension of the award, or termination of the award. It also requires institutions and individuals to notify the NSF if the PI or Co-PI is placed on administrative leave or if any administrative action has been imposed relating to any finding or determination of an investigation of harassing behaviors, including sexual harassment. In addition, behavioral expectations explicitly mentioned in the policy include responsible and accountable comport during the award period at the awardee institutions and online, as well as at conferences, field sites and workshops [24].

The National Institutes of Health (NIH) has recently adopted a similar policy, detailed in Section 239 of the Consolidated Appropriations Act for fiscal year 2022. Like Article X, this law requires grant recipients and their home institutions to report if key personnel are disciplined or placed on leave due to harassment [25].

### Rationale for present study

As there are limited data on the efficacy of Article X and its rollout, it remains unclear whether Article X and its implementation is succeeding and should serve as a model for the other federal agencies and non-governmental funding organizations that are following or may soon follow suit. On the one hand, Article X is promising from a theoretical standpoint. A recurrent critique of the status quo is that universities are disincentivized from dealing with sexual harassment for fear of it impacting their reputation [26, 27]. However, Article X also may have meaningful limitations as concerns have been raised about other initiatives that share certain characteristics with it. In particular, Holland and colleagues have critiqued initiatives around mandatory reporting in the scientific community by insisting that “reporting is not supporting” [28]. Without additional infrastructure, safeguards, and consideration of the larger organizational cultural environments in which policies are implemented such policies may be ineffective and even have unintended negative consequences, including placing additional burdens on survivors and putting them at risk for retaliation [29] It is also necessary to note that mandatory reporting, like sexual harassment itself, may have consequences that differ based on individuals’ different marginalized social identities [30, 31].

To better understand how Article X may impact the common and important problem of sexual harassment in academic science, it is critical to engage with the perspectives of grant award recipients. In light of the high prevalence and need to address sexual harassment in STEM, along with the gap in knowledge about the impact of NSF’s recent policy intervention, the present survey study aimed to understand NSF grantees’ perspectives regarding Article X, including their awareness of the policy and their attitudes regarding its appropriateness. Given how experiences of sexual harassment vary greatly based on personal experiences, gender, and other identities, we also examined how demographic characteristics and past experience with sexual harassment influence those perspectives. Incorporating the viewpoints of grant recipients, who are important stakeholders, is essential to inform NSF’s own policy, the NIH’s implementation of a policy similar to Article X, as well as that of other federal scientific agencies and nongovernmental funders who may be contemplating such policies in the future. We hope these empirical data about NSF grantees, who are scientists in charge of leading their research teams within various institutions, can ultimately provide valuable insight to federal and non-governmental scientific agency leaders and other policy makers interested in implementing and refining policies such as Article X.

## Material and methods

### Participants and recruitment

Between March 5, 2021, and April 16, 2021, a 10-minute online survey about “opinions about recent National Science Foundation policies” was administered via email to a stratified random sample of 700 NSF-funded scientists throughout the United States who led active grants in 2019, of whom 215 responded. In our sampling we prioritized diversity in terms of gender and race. Specifically, we ensured that of the 100 individuals sampled from each of 7 NSF directorates, half appeared to be men and half appeared to be women. We included all those who were likely to be Black and Hispanic individuals and then included Asian grantees up to a total of 30% of the sample, with the remainder being individuals whose names were consistent with non-Hispanic White race-ethnicity (see **S1 File** for further details). We sent an electronic survey using the automated software program Qualtrics with a $10 Amazon electronic gift card incentive (not conditional on response) in the initial email invitation to the identified sample. We used a modified Dillman approach to remind nonrespondents and maximize response rates [32]. Participants were 55.8% women and 38.6% men; 5.6% were gender non-binary, responded to “none of these,” or did not respond. Based on self-report of race and ethnicity (see **S1 File**), we categorized 5.6% as Asian/Asian American/Pacific Islander, 8.4% as Black/African American, 11.6% as Hispanic/Latina(o), 64.7% as White, and 9.8% as other or missing. Of respondents, 86% were both cisgender and heterosexual, 8.8% were LGBTQIA+, and 5.1% did not respond to these items. Sample demographics are summarized in **Table 1**.

**Table 1:** Demographic characteristics of sample.

Variable	Level	Total	Men	Women
		N (%)	n (%)	n (%)
Gender	Man	83 (38.6)	83 (38.6)	
	Woman	120 (55.81)		120 (55.81)
	Gender non-binary	1 (0.47)		
	None of these	2 (0.93)		
	Declined to answer	9 (4.19)		
Racial group	White/Caucasian	139 (64.65)	57 (68.67)	79 (65.83)
	Asian/Asian American/Pacific Islander	12 (5.58)	5 (6.02)	7 (5.83)
	Hispanic/Latina(o)	25 (11.63)	7 (8.43)	18 (15)
	Black/African American	18 (8.37)	10 (12.05)	8 (6.67)
	Other or not reported	21 (9.77)	4 (4.82)	8 (6.66)
Sexual Orientation and Gender Identity Status	Cisgender and heterosexual	185 (86.05)	76 (91.57)	109 (90.83)
	LBGTQIA+	19 (8.84)	7 (8.43)	9 (7.50)
	Decline to answer	11 (5.12)		2 (1.67)
Age	30–39	26 (12.09)	6 (7.23)	20 (16.67)
	40–49	73 (33.95)	37 (44.58)	36 (30)
	50–59	50 (23.26)	16 (19.28)	32 (26.67)
	60+	48 (22.32)	19(22.89)	28(23.34)
	Decline to answer			
Academic rank	Instructor/Assistant Professor	24 (11.17)	8 (9.64)	15 (12.50)
	Associate Professor	58 (26.98)	24 (28.92)	34 (28.33)
	Full Professor	90 (41.86)	40 (48.19)	48 (40.00)
	Non-teaching position/Emeritus	25 (11.63)	9 (10.84)	16 (13.34)
	Other	11 (5.12)	2 (2.41)	7 (5.83)
	Decline to answer	7 (3.26)		
Specialty^a^	CISE/ENG/GEO/OIA/OISE/MPS	108 (50.23)	45 (54.22)	61 (50.83)
	BIO	36 (16.74)	11 (13.25)	24 (20.00)
	EHR/SBE/ERE	63 (29.30)	26 (31.33)	35 (29.17)

^**a**^Computer and Information Science and Engineering (CISE), Geosciences (GEO), Engineering (ENG), Office of Integrative Activities (OIA), International Science and Engineering (OISE), Mathematical and Physical Sciences (MPS), Biological Sciences (BIO), Directorate for Education and Human, Resources (EHR) Social, Behavioral and Economic Sciences (SBE), Environmental Research and Education (ERE).

### Survey instrument

A 49-item questionnaire was developed following best practices in questionnaire design. The survey is comprised of questions on knowledge about the general subject of sexual harassment and policies to address it; attitudes or opinions on the specific policy Article X; personal experiences as measured by the validated Sexual Experiences Questionnaire (SEQ) [33, 34]. We also collected demographics related to gender, race/ethnicity, sexual orientation, and age, as well as background information such as participant seniority and the NSF research area of largest NSF grant received. The entire survey instrument is presented in the **S1 File**.

The SEQ is a validated measure that asks participants to indicate whether they had experienced any of 20 unwanted behaviors in the years from colleagues, superiors, or others at their place of work [33, 34]. Consistent with prior use of the SEQ measure in other studies, participants were instructed to only respond about “unwanted behaviors since March 2020.” We designed the other questions after consideration of the published literature and consultation with subject matter experts within the University of Michigan’s Institute for Research on Women and Gender, its ADVANCE program, and its Center for Bioethics and Social Sciences in Medicine. This included pilot testing and cognitive pretesting of the entire survey instrument with individuals similar to the intended target population, using verbal probing and think-aloud reasoning, to evaluate the final instrument prior to administration. This study was deemed exempt from full review by the University of Michigan institutional review board; participants were provided with a document that included the key elements necessary for informed consent and told in the study invitation that completion of the survey would be taken as their indication of consent to participate.

### Data analysis

#### Quantitative

We provide descriptive statistics as well as bivariable comparisons by identity and a binary variable for whether the respondent had directly experienced any form of sexual harassment since March 2020, as per the SEQ measure, using chi-squared testing for categorical variables and t-tests for continuous scaled measures. Multivariable linear regression models were constructed to evaluate associations between the dependent variable of attitude towards Article X as a continuous measure and the independent variables of race, gender (men versus women), LGBTQIA+ identification (those who selected only man or woman and heterosexual or straight (cisgender and heterosexual category) versus those who selected the nonbinary, transgender, or other category for gender or who selected any other descriptor of sexual orientation), age, and personal experiences with sexual harassment.

#### Qualitative

Open responses to the questions “What concerns do you have with regards to this policy?” and “Please share anything else you wish to provide about your experiences with sexual harassment or relevant policy” were examined through thematic analysis, focusing on additional feedback or context individuals provided about their opinions on Article X that either supplemented or reinforced the fixed response items. The analytic plan proceeded through the six steps outlined by Braun and Clark [35]. Coding was performed independently by two members of the research team (CP and AKG) with training and expertise in qualitative research who then met to establish consensus.

## Results

### Harassment knowledge and experiences

The National Academies defines sexual harassment as “(1) gender harassment (verbal and nonverbal behaviors that convey hostility, objectification, exclusion, or second-class status about members of one gender), (2) unwanted sexual attention (verbal and physical unwelcome sexual advances, which can include assault), and (3) sexual coercion (including when favorable professional or educational treatment is conditioned on sexual activity” (17). Participants appeared aware of what kinds of behaviors constitute sexual harassment, with 91.2% of participants identifying sexist remarks as sexual harassment, 97.2% identifying unwanted sexual attention, and 96.3% identifying sexual coercion as a type of harassment. On the other hand, only 80.0% indicated that crude behaviors constituted sexual harassment.

**Fig 1:**
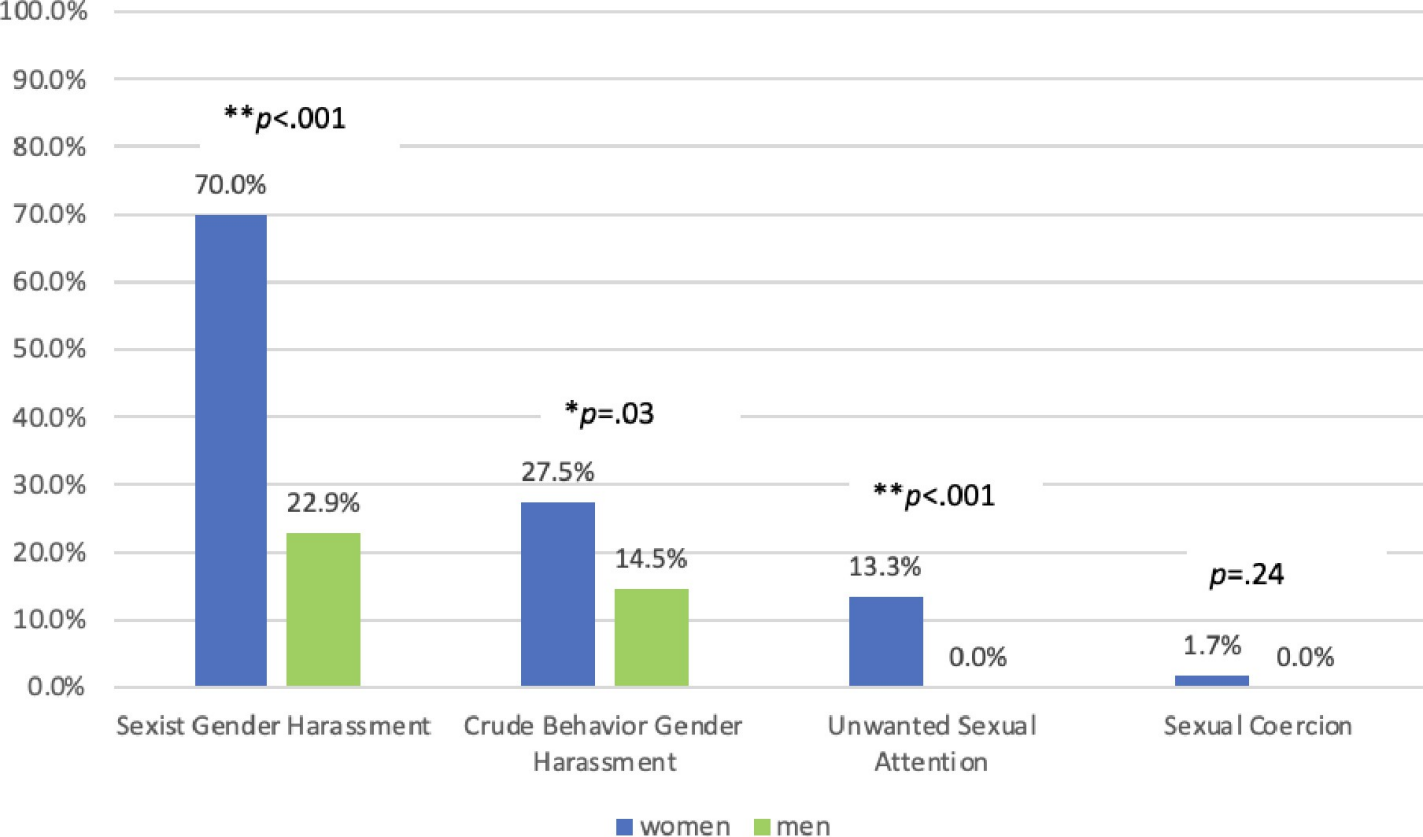
Gender differences in experiences of harassment.

Women were significantly more likely than men to have experienced sexual harassment according to the SEQ. 73.3% of women had experienced harassment compared to only 31.3% of men (p < .001). Sexist gender harassment was the most common form of harassment, affecting 48.8% of all participants. This type of harassment was experienced significantly more by women than men (70.0% compared with 22.9%, p < .001). Crude behavior gender harassment was the second most common form of harassment, impacting 22.0% of participants. It was also more commonly experienced by women than men (27.5% compared with 14.5%, p = .03). Unwanted sexual attention impacted 7.4% of the sample, all of whom were women. There was a statistically significant difference based on gender (13.3% of women compared with 0% of men, p < .001). Sexual coercion was rare, impacting only 2 individuals who were both women; the difference based on gender was not statistically significant given the small numbers indicating having this experience in our sample. These findings are detailed in **Fig 1**.

### Knowledge of Article X

In contrast to participants’ relatively high knowledge about sexual harassment in general, there was a clear lack of knowledge about Article X’s particular details and even its existence. Only 14.0% correctly identified Article X as NSF’s policy on sexual harassment when given a list of options (“Important Notice No. 144: Harassment”; “Article X of the Federal Register: Notification Requirements Regarding Findings of Sexual Harassment, Other Forms of Harassment, or Sexual Assault”; “NSF Office of Diversity and Inclusion ODI Bulletin No. 18–01”; or “I’m not sure”). Regarding knowledge about the details of NSF’s policy only 35.5% were aware of an anonymous reporting mechanism on the NSF website to submit allegations of abuse, fraud, or misconduct and 40.0% were aware that anyone may submit reports or findings of harassment to the NSF via email. However, 65.1% were aware that institutions and individuals must notify the NSF if a principal investigator (PI) or co-principal investigator (Co-PI) is placed on administrative leave or if any administrative action has been imposed relating to any finding or determination of an investigation of harassing behaviors, including sexual harassment. While 91.2% endorsed that these policies were applicable at the awardee’s institution and 87.4% endorsed at field sites for research, 80.5% believed they applied at workshops, 81.9% at conferences, and 76.3% online. Of respondents, 81.4% were aware that the NSF has the ability to remove a PI or co-PI found responsible for harassment from an award, 68.8% believed the NSF could terminate the grant in these circumstances, 67.4% believed they could suspend the grant, and only 33.5% believed they could reduce the funding amount of the award.

### Attitudes toward Article X

**Fig 2:**
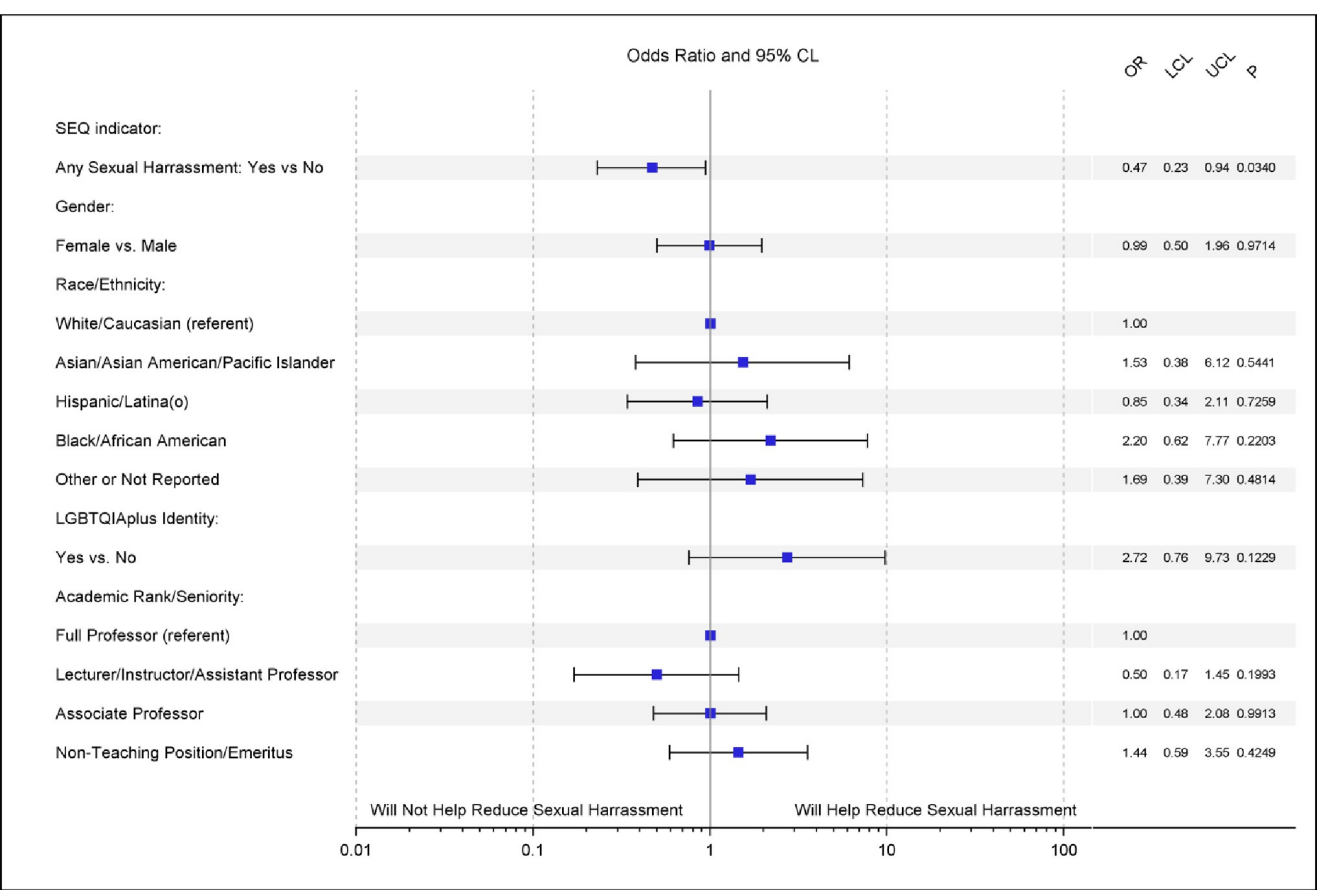
Forest plot of the adjusted associations between the belief that Article X will be “Likely’ or “Extremely likely” to help reduce sexual harassment in the sciences and respondent characteristics. Adjustment covariates were reduced and include gender, race, LGBTQIA+ identity, academic rank, and any sexual harassment experience (SEQ overall indicator).

After reading an explanation of the policy provided in the questionnaire (see **S1 File**), the majority of respondents (63.7%) reported feeling that Article X was likely or very likely to reduce sexual harassment in science. While no demographic factors were associated with this item on bivariable analysis, there were significant differences based on respondent past experience of harassment. On multivariable analysis [**Fig 2**] controlling for race, gender, LGBTQIA+ identification, and seniority, respondents who reported past year sexual harassment were nearly half as likely to believe the policy would help reduce sexual harassment in the sciences compared to those who had not experienced harassment (OR = 0.47, 95% CI:0.23–0.97, p = .034). There was no significant association between gender or any of the other identity characteristics and the perspective of the policy’s likelihood of efficacy.

**Fig 3:**
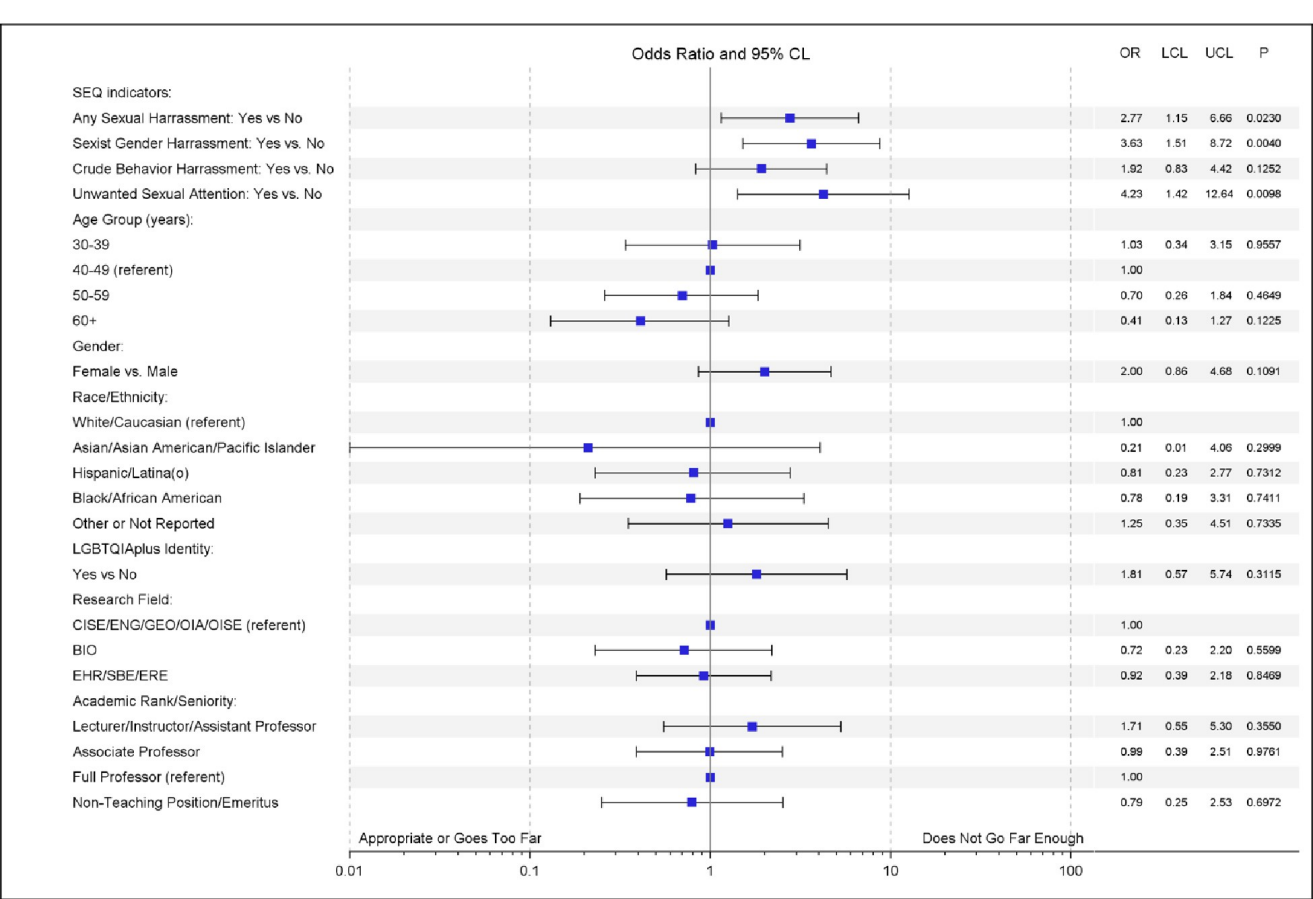
Forest plot of the bivariate associations between belief that the Article X policy “Does not go far enough” and respondent characteristics and the experience of gender harassment. Adjustment covariates were reduced and include gender, race, LGBTQIA+ identity, academic rank, and any sexual harassment experience (SEQ overall indicator).

**Fig 4:**
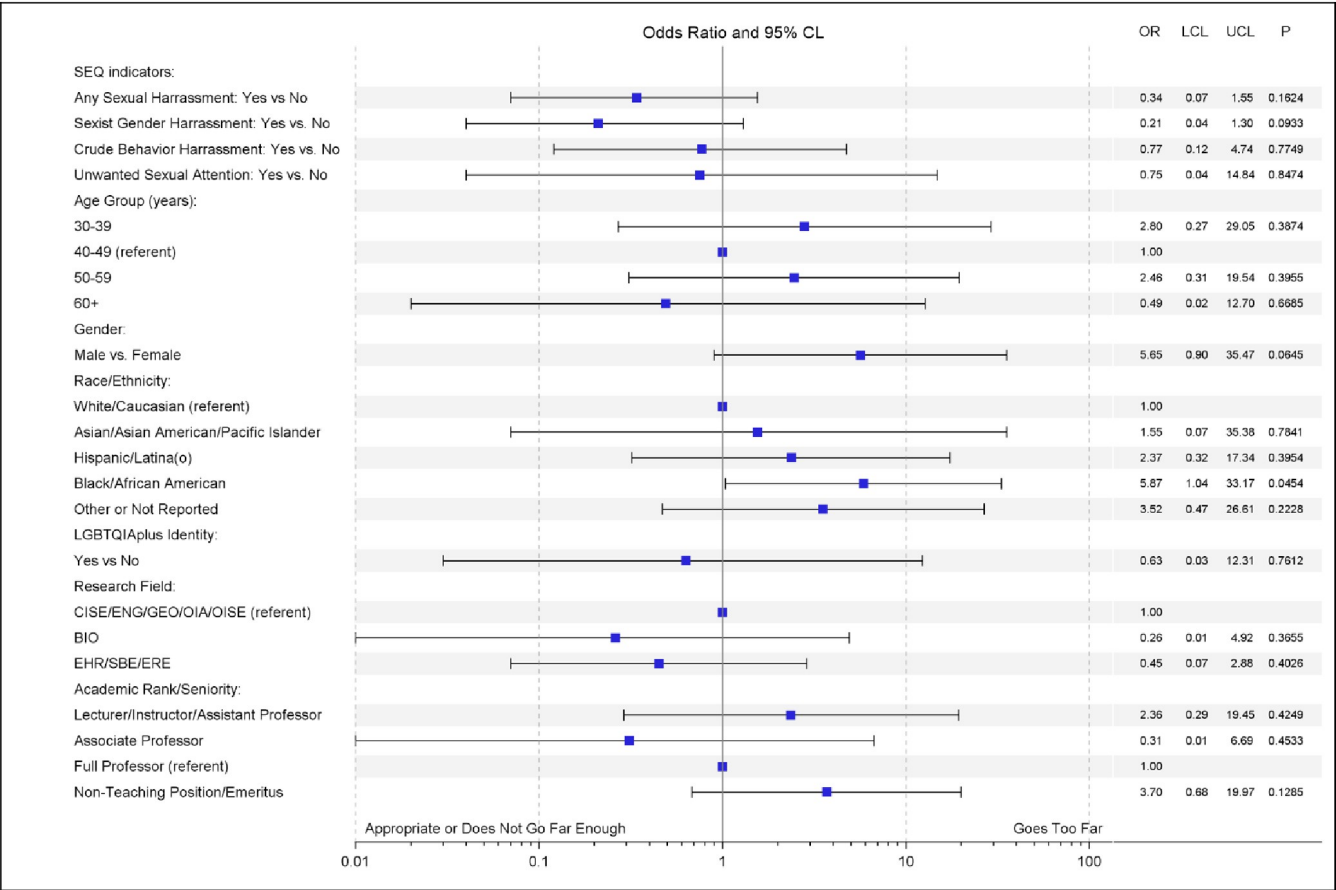
Forest plot of the bivariate associations between belief that the Article X policy “Goes too far” and respondent characteristics and the experience of gender harassment. Adjustment covariates were reduced and include gender, race, LGBTQIA+ identity, academic rank, and any sexual harassment experience (SEQ overall indicator).

When asked how far the policy went in addressing the issue of harassment, 80.5% asserted it was appropriate, 14.2% believed it didn’t go far enough, and only 3.3% thought it went too far. Here we found significant differences based on gender and past experience of harassment. Treating this Likert scale response as a continuous variable [**Table 2**], female gender was associated with a 0.18 (95% CI: 0.03–0.34) lower score (p = .019), suggesting that women were more likely to believe the policy does not go far enough compared to men. The scale was also significantly associated with a respondent experiencing sexual harassment versus none, with an experience of harassment being associated with a 0.21 (95% CI: 0.06–0.36) lower score on the scale (p = .007). When treating the attitudinal measure as a binary variable comparing those who thought the policy failed to go far enough versus who did not [**Fig 3**], having directly experienced harassment was associated with a greater odds of endorsing the policy failed to go far enough (OR = 2.77, 95% CI:1.15–6.66, p = .023). When treating the attitudinal measure as a binary variable comparing those who thought the policy went too far versus those who did not [**Fig 4**], the only variable significantly associated with believing that the policy goes too far was race, with African American respondents being nearly six times as likely to endorse this belief (OR = 5.87, 95% CI:1.04–33.17, p = .045).

**Table 2:** Bivariate regression estimates for the association with B2 continuous “how far policy goes” scale (item asking respondents to rate their perceptions of the policy going not far enough to going too far).

Parameter_	Estimate	LowerCL	UpperCL	PairwiseP	GroupP
Age					
30–39	0.019230769	-0.226863247	0.265324786	0.8777	0.3384
40–49 (reference)	0.000000000	.	.	.	.
50–59	0.150000000	-0.048002012	0.348002012	0.1368	.
60+	0.145833333	-0.054588345	0.346255012	0.1529	.
Gender					
Women vs Men	-0.184552846	-0.338357797	-0.030747894	0.0189	0.0189
Race					
White/Caucasian (reference)	0.000000000	.	.	.	0.6362
Asian/Asian American/Pacific Islander	0.173913043	-0.151509896	0.499335983	0.2933	.
Hispanic/Latina(o)	0.013913043	-0.221113523	0.248939610	0.9072	.
Black/African American	0.173913043	-0.097055704	0.444881791	0.2072	.
Other or Missing	-0.002557545	-0.280486981	0.275371891	0.9855	.
LGBTQIA+ Identity					
LGBTQIA+ vs Not	-0.069165059	-0.328794690	0.190464572	0.6000	0.6000
NSF Science Area					
BIO	0.038681205	-0.169918562	0.247280971	0.7150	0.8397
CISE/ENG/GEO/OIA/OISE	0.000000000	.	.	.	.
EHR/SBE/ERE	0.048537835	-0.124262490	0.221338161	0.5803	.
Academic Rank					
Lecturer/Instructor/Assistant Professor	-0.116425121	-0.368180074	0.135329832	0.3629	0.5018
Associate Professor	-0.048538012	-0.230937644	0.133861621	0.6004	.
Full Professor (reference)	0.000000000	.	.	.	.
Other	0.088888889	-0.123600253	0.301378030	0.4104	.
Personal Experience of Sexual Harassment (yes to any item on SEQ)					
Yes vs No	-0.205982906	-0.355781866	-0.056183946	0.0073	0.0073

Respondents also endorsed a number of concerns about the policy. Only 1.4% were worried that it undermines local institutional authority, 2.3% that it conflicts with other regulations or laws, 4.2% that the policy was unclear, and 8.8% that it compromises the privacy of survivors of harassment. On the other hand, 14.9% endorsed being concerned that it would jeopardize funding at their institution, 20.0% that it expands the risk of retaliation against complainants, and 21% that it could damage the careers of complainants. In addition, 48.4% agreed that it could damage the careers of those accused of harassment.

There were significant differences based on respondents’ past experiences of harassment and gender. Respondents endorsing experiencing sexual harassment in the year prior to survey administration were more likely to support the concern that Article X could damage the careers of complainants (OR = 2.38, 95% CI:1.15–4.92, p = .019). The opposite association was seen for the concern that Article X could damage the careers of those accused of harassment; those reporting any lived experience of sexual harassment were significantly less likely to endorse that particular concern (OR = 0.41, 95% CI 0.23–0.71 p = .0018). Men were also more likely to agree that Article X could be damaging to the careers of the accused (OR = 1.77 95% CI: 0.86–3.74 p = .0499). On the other hand, women were significantly more likely to endorse that the policy could compromise the privacy of survivors (OR = 0.20, 95% CI: 0.05–30.78 p = .020) or increase the risk of retaliation against the complainant (OR = 0.41, 95% CI: 0.19–0.89 p = .024).

### Qualitative findings

Overall, 92 participants provided substantive answers to one or both open-response questions. Of these, 19 individuals offered positive feedback regarding Article X. This feedback tended to be general appreciation that NSF was using its position to take a stand against harassment. One wrote, “NSF can play a big role in making it clear that inappropriate behavior will not be tolerated,” while another simply stated, “Great that NSF is getting involved to monitor abuses in this area!” Two individuals explicitly mentioned that they believed targeting institutional funding was a key lever for change. For example, one noted, “NSF’s policies against discrimination and harassment are critically important in changing the culture around discrimination of under-represented groups. Research funding is a major motivator for behavior change.”

A total of 44 individuals expressed concerns about Article X in their responses. The majority of these concerns (n = 26) were related to enforcement of the policy, particularly at the institutional level. One wrote that “[t]he policy wholly depends on home institutions investigating and registering findings, but this is extremely difficult for home institutions to do well when researchers are using remote facilities or carrying out remote fieldwork. Thus, instances will still be under-reported and poorly enforced.” Another explained that “policy success is dependent on implementation and use. An ideal policy fails if its protections and penalties are never used.” Addressing these concerns, one scientist suggested that the policy “should state institution consequences for failure to report.” Relatedly, five mentioned that the university had little incentive to report and may in fact be dis-incentivized to investigate harassment as a result of the policy, which “could provide incentives for university admin to cover up harassment so the institution doesn’t lose funding.” Eight mentioned that they had been unaware of the policy or felt that it had not been sufficiently publicized. Five expressed concerns about how the policy would negatively impact the accused, such as one who wrote “Could damage the careers of those falsely accused of harassment.”

Eleven scientists noted that the policy did not go far enough or needed to be expanded to other forms of harassment and sexism such as “bullying” or other “types of demeaning behavior women in STEM are subjected to.” Four suggested the policy should give institutions more specific guidance on how to comply and consequences for not complying; two suggested harsher penalties were needed. Additionally, three respondents mentioned a need for measures that protected the survivors of harassment or supported their ability to report. As one explained: “The problem is that it does not create a new, confidential method for reporting in the first place. The problem is not that harassers are not punished enough; the problem is that they are never found guilty of harassment in the first place, because the risks to complainants are too high. There needs to be a new way for the NSF to listen.”

It is also worth noting that a number of participants commented in the open response questions on harassment experiences in their own lives or at their own institutions preceding the 12-month time frame that the survey questions asked participants to consider. Specifically, 22 mentioned that the virtual environment due to COVID-19 social distancing had altered their own exposures to harassment (typically making it less likely than in the past to have encountered such behaviors in person) and might influence the quantitative results about harassment prevalence. Six expressed a feeling that positive organizational culture shifts were occurring or had occurred over the course of their careers in terms of harassment. However, 25 described the pervasiveness of problematic organizational cultures characterized by sexist behavior and uneven power dynamics.

## Discussion

Overall, participants had positive perspectives on Article X and, in particular, an appreciation of the NSF using its platform and financial leverage to address this issue. An overwhelming majority of the respondents believed that the policy would have an impact on sexual harassment in science. Additionally, most believed the policy’s scope was appropriate, that it neither went too far nor not far enough. Given the challenging and often charged nature of discussions around harassment, these assessments suggest that Article X might be used as a positive model for future initiatives, pending future evaluations of its efficacy.

Still, the policy was not without critique. Notably there was a clear lack of knowledge about the policy among scientists in our study (PIs listed as having active grant awards in 2019) who arguably would have been exposed to the publicity of this policy when it was implemented the year prior, which suggests more education and publicity accompanying such interventions is needed. Very few participants were able to correctly name the policy or its most salient details. Additionally, in free response questions, individuals explicitly noted that they had not heard about the policy or that this survey was where they had first learned about what it entailed.

More substantively, echoing previous discussion about how “reporting is not supporting” [28], many respondents had a hunger for other measures to protect survivors and to take into account organizational cultures when implementing the policy. Given these findings and the insights of scholars who have studied the impact of policy on intended outcomes [36], pursuing a deeper understanding of the interactions between implementation of Article X and organizational culture may constitute a particularly valuable endeavor for NSF in future research. Some noted too that there appeared to be gaps in the policy, particularly around how institutions would be held accountable for complying with Article X, especially given that some might have financial incentives not to report harassment in order to avoid losing grant funding. Without enforcement and institutional compliance, there was a perception that the initiative would, at best, fall short of its potential and, at worst, potentially harm targets of harassment who chose to report.

Another key finding is that past-year harassment experiences appeared to be the most important factor in shaping perceptions of the potential efficacy of interventions such as Article X, more than any particular identity traits. It was also apparent that gender plays a role in influencing perceptions about whether or not such interventions are sufficient. Gender also had an impact on whether the biggest concerns about the policy were about the well-being of the accused versus the complainant. The alignment of male respondents with concerns for the accused and female respondents with concerns for the complainant align with previous studies about who is more likely to perpetrate harassment versus be a target of it [11–13].

Overall racial background did not appear to impact participants’ views of or knowledge of Article X. However, one exception was that Black respondents were more likely to believe that the policy goes too far. This finding may reflect concerns that Black men may be at greater risk of being falsely accused of harassment, despite abundant evidence that false accusations are rare [37, 38]. Evidence does suggest that Black men are more likely to be convicted if accused compared to their white counterparts [39]. Given these findings, it appears that it would be helpful for the policy to include language acknowledging the possibility of race-related bias and how fairness will be ensured in the policy’s implementation.

It is also interesting to note that age did not play a significant role in attitudes towards the policy. Previous work suggests generational differences in the perception of sexual harassment, including perceptions of female complainants’ credibility [40]. On the other hand, though, other studies have found limited generational differences related to perceptions of harassment [41]. As such, there appears to be a need for more research in this area to better understand the impact of age on views of sexual harassment in a post-#MeToo era.

Finally, our findings suggest the pervasiveness of harassment even in an increasingly virtual world. In spite of concerns expressed by participants about potentially artificially deflated numbers due to the timing of the survey during the COVID-19 pandemic, the prevalence of harassment reported by these participants was not notably different from pre-COVID surveys nor from other surveys that were conducted during the pandemic [42, 43].

### Strengths and limitations

The novelty of this study is one strength as, to the authors’ knowledge, this is the first research to explore knowledge and perspectives of Article X or other federal sexual harassment policies within STEM. Additionally, we had a fairly diverse sample which allowed us to examine differences in opinion and experience based on gender and race. One weakness of the study was its timing during COVID-19, which may have led to lower reports of sexual harassment experiences than when in-person interaction was occurring regularly, although there is some evidence that the lack of in-person interactions did not decrease the incidence of the most common form of sexual harassment, gender harassment, which can easily be perpetrated via virtual interactions [42, 43]. Reassuring regarding this possible weakness (though generally un-reassuring), our results reveal substantial rates of sexual harassment in this sample that appear comparable to past studies conducted before the pandemic. This suggests that harassment was still prevalent during social distancing and that more research should be initiated to better understand how it occurred and the ways in which it differed from pre-pandemic harassment experiences. Additionally, our sample was modest in size, which limited our ability to conduct multivariable adjusted analyses. Only one participant identified as non-binary, limiting our ability to explore the nuances of gendered harassment experiences and perspectives beyond the binary of men versus women.

Finally, it is important to note that our study is only a first step towards understanding whether policies such as Article X will succeed in achieving their intended outcomes of reducing the frequency and consequences of harassment in science. Existing literature suggests that organizational and professional culture is a primary determinant of sexual harassment, and policy implementation may not directly alter culture [29, 36]. Culture shapes how policies are interpreted and applied [36]. Therefore, additional research will be essential to evaluate the ultimate impact of Article X and other similar policies and how their intended outcomes might be optimized, including qualitative research on the interaction between policy and organizational culture.

## Conclusion

Our study examined scientists’ knowledge and attitudes about the NSF’s sexual harassment policy, Article X. We found that participants responded positively to this policy overall and believed it would have an impact on gender harassment in STEM. Nevertheless, our findings also highlighted deficiencies in the dissemination of information about Article X, as many respondents were not aware of the policy nor its details before participating in our study. Additionally, some individuals, particularly those with past experiences of harassment, believed that the policy did not go far enough. Participants also raised critical questions about the institutional enforcement of these policies and the existence of sufficient protections for survivors and complainants. Congress recently asked for an update on NSF’s sexual harassment policy, and we believe these data provide information from one key stakeholder cohort to inform action in this rapidly evolving area. Our work has clear implications for the NSF’s continued evaluation of the efficacy of its efforts as well as for other federal agencies and nongovernmental funders who are implementing or considering similar policies, highlighting in particular the need to consider interactions between policy and organizational culture to foster meaningful positive change.

## Supporting information

S1 File Supplementary materials. Supplementary Appendix 1: Participants and Recruitment Strategy. Supplementary Appendix 2: Survey Instrument.
(PDF)
