# Supplementary material for: National science foundation grant awardees’ perspectives on Article X and sexual harassment in science
Source: PLoS One. 2024 Apr 30;19(4):e0300762. doi: 10.1371/journal.pone.0300762 (PMC11060523; doi:10.1371/journal.pone.0300762)
Supplement: S1 File — Supplementary Appendix 1: Participants and Recruitment Strategy. Supplementary Appendix 2: Survey Instrument. (PDF) [file pone.0300762.s001.pdf]

Supplementary Materials

Supplementary Appendix 1: Participants and Recruitment Strategy

In order to generate our study sample we identified 12,038 scientists who had active grants in 2019 in the publicly available databases present on the NSF.gov website, one year after Article X was fully implemented. We used R-Studio software and created syntax to gather necessary information to create a database to allow for stratified random sampling. To create an appropriate framing sample, we used an application programming interface (API) (specifically an R package for “gender” and “wru” for race) which generated ethnicity and gender estimates for the NSF grant awardees in our database. Our overall estimates for the entire 12038 population using this API was approximately 75% white, 20% Asian/Asian American, and 5% other races and ethnicities underrepresented in medicine (URiM) and science.

We randomly selected 100 participants from each NSF directorate (Biological Sciences (BIO), Computer and Information Science and Engineering (CISE), Geosciences (GEO), Engineering (ENG), Mathematical and Physical Sciences (MPS), Social, Behavioral and Economic Sciences (SBE), Directorate for Education and Human Resources (EHR)) with random selection after stratification by variables of interest (gender and race) to power the study to detect meaningful differences among population subgroups in aggregate. We sampled gender first to generate a pool that was approximately evenly distributed between men and women in each of the 7 NSF directorates. We then oversampled for under-represented minorities by including all individuals who appeared to be URiM in each directorate. Finally, then we included Asian grantees up to a total of 30% of the sample in each directorate, with the remainder being individuals whose names were consistent with non-Hispanic White race-ethnicity. Although we aimed for this 70/30 split between non-Hispanic White and Asian or other URiM in the overall sample, the numbers of URiM and Asian individuals in some directorates were too low to achieve this ratio. Our sample, accordingly, was further skewed towards non-Hispanic White individuals. We chose these percentages as they roughly correlated to the distribution in the overall 2019 grant awardee dataset, accounting for the fact that there may have been some error in the race and gender categories due to the use of an application programming interface. After

identifying a preliminary sample through this process, 5 research assistants manually conducted an internet search for institution-specific websites of each of the 700 individuals selected using publicly available information online in order to provide a second check of the API's gender and race categorizations. Random substitutions were made when errors were found, but we acknowledge there was still likely a level of human error in creating estimations of race and gender social identities from publicly available websites. We accepted this as we designed the study to facilitate self-reported social identities for our analysis, which would supersede these initial qualitative estimations once the surveys had been completed. In designing the initial sampling process we prioritized being able to detect meaningful differences between men and women and to provide descriptive summary statistics based on directorates and on race (noting we might not have had enough power to detect differences in the race category depending on the response rate).

In the survey itself we asked participants to self-report race/ethnicity using the following categories: White/Caucasian, Asian/Asian American/Pacific Islander, Hispanic/Latina(o), Middle Eastern, Native American/American Indian, Black/African American. Individuals who responded to more than one category were placed in the latter category (according to the order listed here). For example, an individual who marked both White/Caucasian and Hispanic/Latina(o) was categorized as Hispanic/Latina(o). Middle Eastern and Native American/American Indian were combined into an "other" category for analysis given the small number of responses in these categories. There were also two write-in options: "multiracial/ethnic" and "None of these categories describes me." The answers of the 4 respondents to these questions were sorted into the most relevant of the categories.

To administer the study, we sent an electronic survey using the automated software program Qualtrics with a $10 Amazon electronic gift card incentive in the initial email invitation to the identified sample. Our initial email, which was sent on March 5, 2021, provided basic information and served as a cover letter, with an attached consent information document. Completion of the survey was taken to indicate the participant's consent (waiver of written consent was provided by the University of Michigan's IRB). We used a modified Dillman approach to remind nonrespondents and maximize response rates given the timing of the survey administration during the COVID-19 pandemic. Reminder emails were sent every week for 3

weeks to respondents with incomplete surveys and to non-respondents, followed by a reminder phone call and an email afterwards (for a total of up to 6 reminders) to assist the process of maximizing survey completion. Our last reminder occurred on April 2, 2021, after which the survey was left open for participants to complete during an additional 2 weeks. Ultimately, 215/700 respondents (30.71%) completed surveys and constituted the analytic sample. This response rate is comparable to other studies of professionals.

Supplementary Appendix 2: Survey Instrument

We took inspiration from Cabana's conceptual model [1] to consider the barriers to adherence to practice guidelines (for physicians) in relation to behavior change, specifically highlighting the importance of separately assessing knowledge and attitudes. Assessing knowledge was important as theoretically these scientists were to have known about Article X; assessing attitudes was also important as it illuminated policy-relevant perspectives of key stakeholders.

References

1. Cabana MD, Rand CS, Powe NR, Wu AW, Wilson MH, Abboud PA, Rubin HR. Why don't physicians follow clinical practice guidelines?: A framework for improvement. JAMA. 1999 Oct 20;282(15):1458-65.

Section A: Content Knowledge

Science and Policy: A Survey of National Science Foundation Grant Awardees

<u>Section A: Content Knowledge</u>
First, we would like to assess your understanding of sexual harassment and existing policies. Please answer the following questions to reflect your understanding and knowledge.

1. Sexual harassment is comprised of the following: (Check all that apply)

- ☐ Sexist remarks
- ☐ Crude behaviors
- ☐ Unwanted sexual attention
- ☐ Sexual coercion
- ☐ I'm not sure

2. The National Science Foundation (NSF) policy on sexual harassment is known as: (Check all that apply)

- ☐ Important Notice No. 144: Harassment
- ☐ Article X of the Federal Register: Notification Requirements Regarding Findings of Sexual Harassment, Other Forms of Harassment, or Sexual Assault
- ☐ NSF Office of Diversity and Inclusion ODI Bulletin No. 18-01
- ☐ I'm not sure

3. An anonymous reporting mechanism exists on the NSF website to submit allegations of abuse, fraud, or misconduct.

- ○ Yes

- ◯ No
- ◯ I'm not sure

4. Anyone may submit reports or findings of harassment to the NSF via email.

- ◯ Yes
- ◯ No
- ◯ I'm not sure

5. Institutions and individuals must notify the NSF if a principal investigator (PI) or Co-Principal investigator (Co-PI) is placed on administrative leave or if any administrative action has been imposed relating to any finding or determination or an investigation of harassing behaviors, including sexual harassment.

- ◯ Yes
- ◯ No
- ◯ I'm not sure

6. Behavioral expectations explicitly mentioned in the policy include responsible and accountable comportment during the award period at the following locations (check all that apply):

- ☐ Awardee Institutions
- ☐ Online
- ☐ At conferences
- ☐ At field sites for research
- ☐ At workshops
- ☐ None of these

7. If found responsible for harassment, the NSF now has the ability to (check all that apply):

- ☐ Remove the PI or Co-PI from an award
- ☐ Substitute the PI or Co-PI from an award
- ☐ Reduce the funding award amount of the grant
- ☐ Suspend the grant

- [ ] Terminate the grant
- [ ] None of these

Section B: Policy

Section B: Policy
On October 21, 2018, the **National Science Foundation implemented Article X** as published in the Federal Register. Article X seeks to leverage the influence deriving from NSF's control of substantial grant funding to contribute to efforts **to address the problem of persistent and pervasive sexual harassment** in the sciences.
It provides **clear potential consequences for NSF awardees who have been found responsible for harassment, including substitution or removal of the principal investigator or co-principal investigator from the award, reduction in the award funding amount, suspension of the award, or termination of the award**. It also **requires institutions and individuals to notify the NSF** if the PI or co-PI is placed on administrative leave or if any administrative action has been imposed relating to any finding or determination or an investigation of harassing behaviors, including sexual harassment. In addition, behavioral expectations explicitly mentioned in the policy include **responsible and accountable comportment during the award period at the awardee institutions, online, and at conferences, field sites and workshops**.

1. Do you think Article X will help reduce sexual harassment in science?

- ○ Not at all likely
- ○ Unlikely
- ○ Likely
- ○ Extremely Likely

2. What do you think about this policy? On a scale of 1-5, please select one:

- ○ 1 Does not go far enough
- ○ 2
- ○ 3 Appropriate
- ○ 4
- ○ 5 Goes too far

3. What concerns do you have with regards to this policy? Check all with which you agree.

This policy:

- [ ] is unclear
- [ ] undermines local institutional authority
- [ ] conflicts with other regulations or laws
- [ ] compromises the privacy of survivors of sexual harassment
- [ ] expands risk of retaliation against complainants
- [ ] could damage the careers of complainants
- [ ] could damage the careers of those accused of harassment
- [ ] jeopardizes funding at my institution
- [ ] I have no concerns about this policy
- [ ] Other: ______

Section C

Section C: Personal Experiences
Now we will ask about your experiences in the LAST YEAR. These questions ask about a series of UNWANTED behaviors that you may have experienced when you were interacting with staff, students, and faculty, both on and off campus, since March 2020, at your institution. **Thinking about UNWANTED behaviors SINCE MARCH 2020, how often have staff, students, or faculty**:

	Never	Once or Twice	Sometimes	Often	Many Times
1. Mistreated, slighted, or ignored you because you are a woman or man?	○	○	○	○	○
2. Made offensive sexist remarks (for example, suggesting that people of your sex are not suited for the kind of work you do)?	○	○	○	○	○
3. Put you down or been condescending to you because of your sex?	○	○	○	○	○
4. Displayed or distributed stories, pictures, or words that insult or disrespect women generally?	○	○	○	○	○
5. Displayed or distributed sexually explicit stories, pictures, or pornography?	○	○	○	○	○
6. Told sexual stories or dirty jokes?	○	○	○	○	○
7. Tried to get you in a conversation about sex?	○	○	○	○	○
8. Made offensive remarks about your appearance, body, or sexual activities?	○	○	○	○	○

	Never	Once or Twice	Sometimes	Often	Many Times
9. Made gestures or used body language of sexual nature that embarrassed or offended you?	○	○	○	○	○
10. Tried to start a romantic relationship with you after you told the person that you didn't want the relationship?	○	○	○	○	○
11. Continued to ask you for dates, drinks, dinner, etc., even though you said "no"?	○	○	○	○	○
12. Stared or looked at you in a sexual way?	○	○	○	○	○
13. Intentionally touched in any way your thigh, breast, butt, or genitals?	○	○	○	○	○
14. Touched another part of your body in a way that suggests sexual interest?	○	○	○	○	○
15. Tried to touch, fondle, kiss, or grope you?	○	○	○	○	○
16. Exposed or sent pictures of their genitals to you?	○	○	○	○	○
17. Offered you something you wanted at work in exchange for doing something sexual?	○	○	○	○	○
18. Implied that you would receive a professional reward if you did something sexual?	○	○	○	○	○
19. Made you worry that you might be treated badly if you did not do something sexual?	○	○	○	○	○
20. Treated you badly for refusing to do something sexual?	○	○	○	○	○

21. Upon reflection, have you yourself engaged in any of the behaviors described above in the last year that might have been unwanted by others?

- ○ Never
- ○ Once or Twice
- ○ Sometimes
- ○ Often
- ○ Many Times

Section D

Section D: Personal characteristics

1. Please indicate which of the following best describes your current position at your institution

- Lecturer
- Instructor
- Assistant Professor
- Associate Professor
- Full Professor
- Other

2. Please indicate your primary department

Anthropology
Astronomy
Atmospheric sciences
Biochemistry
Biology
Chemistry
Cognitive and Learning science
Computer science
Economics
Education

Please enter your primary department

3. What is your field of study?

Anthropology
Astronomy
Atmospheric sciences
Biochemistry
Biology
Chemistry
Cognitive and Learning science
Computer science
Economics
Education

Please enter your field of study

[ ]

4. What was your terminal degree? (check all that apply)

- ☐ BA
- ☐ BS
- ☐ MS
- ☐ MPH
- ☐ MSc
- ☐ PhD
- ☐ DPhil
- ☐ MD
- ☐ DO
- ☐ ScD
- ☐ JD
- ☐ MBA
- ☐ Other [ ]

5. In what year did you complete your terminal degree?

[ ]

6. What is your age?

[ ]

7. Were you born in the US?

- ○ Yes
- ○ No

8. Is English your native language?

- Yes
- No

9. Which of the following racial/ethnic categories describe(s) you? Mark all that apply.

- White/Caucasian
- Black/African American
- Asian/Asian American/Pacific Islander
- Middle Eastern
- Native American/American Indian
- Hispanic/Latina(o)
- Multiracial/Multiethnic (please describe) ______
- None of these categories describe me. I identify as (please describe) ______

10. Which of the following best describes your gender identity?

- Woman
- Man
- Non-binary
- Trans Man
- Trans Woman
- None of these categories describe me. I identify as (please describe) ______

11. Which of the following best describes your current sexual orientation?

- Heterosexual
- Lesbian
- Gay
- Bisexual
- Pansexual
- Queer

- ○ Asexual
- ○ None of these categories describe me. I identify as (please describe) ________

12. Please indicate which of the following best describes your role on the largest NSF grant you have ever received?

- ○ Principal Investigator
- ○ Co-Principal Investigator
- ○ Co-Investigator

13. Please indicate the total dollar amount of your largest NSF grant award: (An estimate is fine)

____

14. Please select the corresponding NSF research area for this award:

- ○ Biological Sciences (BIO)
- ○ Computer and Information Science and Engineering (CISE)
- ○ Education and Human Resources (EHR)
- ○ Engineering (ENG)
- ○ Geosciences (GEO)
- ○ Mathematical and Physical Sciences (MPS)
- ○ Social, Behavioral and Economic Sciences (SBE)
- ○ Environmental Research and Education (ERE)
- ○ Integrative Activities (OIA)
- ○ International Science and Engineering (OISE)

15. Please indicate which year your NSF grant funding was active: (Select all that apply, for any grant funded by the NSF)

- ☐ 2017
- ☐ 2018
- ☐ 2019

- ☐ 2020
- ☐ 2021

16. How many trainees have you mentored **in the past year**? Please include students, graduate students, post-doctoral fellows, residents, and/or fellows. (An estimate is fine)

17. How many trainees have you mentored **over the span of your entire career**? Please include students, graduate students, post-doctoral fellows, residents, and/or fellows. (An estimate is fine)

18. Have you witnessed trainees (students, post-doctoral fellows, residents, and/or fellows) engaging in harassing behavior in your presence in person over the past year?

- ○ Yes
- ○ No
- ○ I'm not sure

19. Have you witnessed trainees (students, post-doctoral fellows, residents, and/or fellows) engaging in harassing behavior in your presence online during virtual meetings over the past year?

- ○ Yes
- ○ No
- ○ I'm not sure

Section E

Section E: Final Thoughts Please share anything else you wish to provide about your experiences with sexual harassment or relevant policy. All responses will be fully anonymized upon receipt so please feel free to share openly.

Thank you for taking the time to complete our survey. Your responses are greatly appreciated.

After you submit this survey, you will be redirected to a separate survey question about your interest in participating in future interviews.

Powered by Qualtrics
